# Supplementary material for: Yiai Fuzheng decoction inhibits triple-negative breast cancer by remodeling the immune microenvironment
Source: Front Immunol. 2025 Sep 30;16:1615631. doi: 10.3389/fimmu.2025.1615631 (PMC12518410; doi:10.3389/fimmu.2025.1615631)
Supplement: Supplementary file 2 [file DataSheet1.zip › Supplementary File 2/Treatment/D_vs_H/02_PCA/Uni_Group/D_PCA_PC12_Scoreplot.pdf]

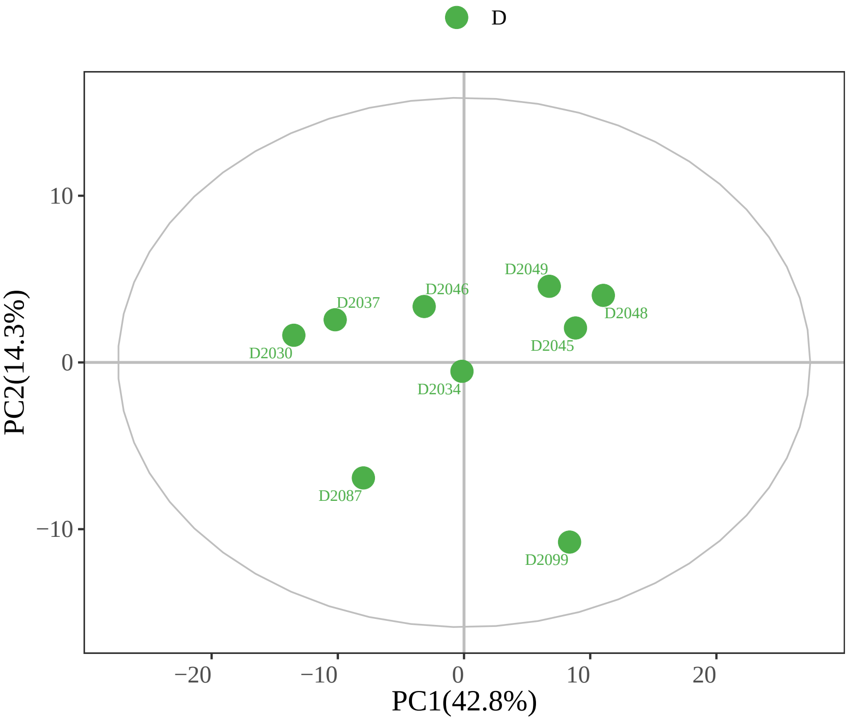

D
D2049
D2046
D2037
D2048
D2045
D2030
D2034
D2087
D2099
10
0
−10
−20
−10
0
10
20
PC1(42.8%)
PC2(14.3%)
